# Parkinsonian neurotoxicants impair the anti-inflammatory response induced by IL4 in glial cells: involvement of the CD200-CD200R1 ligand-receptor pair

**DOI:** 10.1038/s41598-020-67649-4

**Published:** 2020-06-30

**Authors:** Neus Rabaneda-Lombarte, Lucas Blasco-Agell, Joan Serratosa, Laura Ferigle, Josep Saura, Carme Solà

**Affiliations:** 1 Department of Cerebral Ischemia and Neurodegeneration, Institut d’Investigacions Biomèdiques de Barcelona (IIBB)-Consejo Superior de Investigaciones Científicas (CSIC), Institut d’Investigacions Biomèdiques August-Pi i Sunyer (IDIBAPS), c/Rosselló 161, 6th Floor, 08036 Barcelona, Spain; 2 Biochemistry and Molecular Biology Unit, School of Medicine, University of Barcelona, IDIBAPS, Barcelona, Spain

**Keywords:** Cell biology, Neuroscience, Diseases, Pathogenesis, Risk factors

## Abstract

Exposure to pesticides such as rotenone is a risk factor for Parkinson’s disease. Dopaminergic neurons are especially sensitive to the toxicity of compounds that inhibit the mitochondrial respiratory chain such as rotenone and 1-methyl-4-phenylpyridinium (MPP+). However, there is scarce information on their effects on glia. To evaluate whether these neurotoxicants affect the immune response of glia, primary mouse mixed glial and microglial cultures were treated with interleukin (IL) 4 in the absence and presence of MPP+ or rotenone. Using qRTPCR or western blot, we determined the expression of anti-inflammatory markers, the CD200R1 microglial receptor and its ligand CD200, and genes regulating glycolysis and oxidative metabolism. ATP and lactate levels were additionally determined as an index of cell metabolism. Microglial phagocytosis was also evaluated. MPP+ and rotenone clearly abrogated the IL4-induced expression of anti-inflammatory markers in mixed glial cultures. CD200 and CD200R1 expression and microglia phagocytosis were also affected by the neurotoxicants. Changes in the mRNA expression of the molecules regulating glycolysis and oxidative metabolism, as well as in ATP levels and lactate release suggested that metabolic reprogramming in response to MPP+ and rotenone differs between microglial and mixed glial cultures. These findings support the hypothesis that parkinsonian neurotoxicants may impair brain immune response altering glial cell metabolism.

## Introduction

Exposure to certain environmental agents can be a risk factor for the development of neurological disorders and other human chronic diseases^1^. In the case of Parkinson’s disease, exposure to compounds that inhibit the mitochondrial respiratory chain, such as the pesticide rotenone (functional analogue of the parkinsonian neurotoxicant 1-methyl-4-phenyl-1,2,3,6-tetrahydropyridine, MPTP) is a risk factor^2,3^. Most of the studies investigating the effect of these toxicants on the brain have focused on neuronal cells^4^. However, as glial cells are potentially involved in the development of Parkinson’s disease^5,6^, the effects of these agents on glial cells merit to be studied. Nevertheless, studies on the direct effects of these neurotoxicants on glial cells are scarce, and controversial results have been reported for MPTP (or the MPTP active metabolite1-methyl-4-phenylpyridinium, MPP+)^7–11^ and rotenone^11–15^.

Homeostasis in the brain parenchyma is maintained by the combined action of neurons and glial cells. Changes in the brain microenvironment may result in direct changes in neuronal and glial cell function. In addition, due to neuron-glia crosstalk, changes in neuronal function may secondarily affect glial cell function and vice versa^16^. Consequently, external stimuli that play a role in the development of neurological diseases may affect both neuronal and glial cell function.

Glial cells, mainly microglia, are the main representatives of the endogenous immune system in the central nervous system (CNS). In response to changes in brain function, microglial cells, the resident macrophages in the CNS, become quickly activated and undergo morphological and functional changes in order to recover brain homeostasis^17,18^. Depending on the triggering stimulus, microglial activation involves phenotypes that, by analogy to peripheral macrophages, range from the classical activation or M1-like phenotype, which is mainly pro-inflammatory and potentially neurotoxic, to the alternative activation or M2-like phenotype, which is mostly anti-inflammatory and neuroprotective. However, there is general consensus that the status of activated microglia is more complex than this, with purely M1 or M2 polarization not existing, but rather a continuum of these two functional states of polarization and the coexistence of multiple microglial phenotypes^19,20^. Nevertheless, in the experimental in vitro models used to study glial cell function, stimuli like LPS or IFN-γ are used to induce a pro-inflammatory response (modeling an M1-like phenotype), while stimuli like IL4 are used to induce an anti-inflammatory response (modeling an M2-like phenotype). The extent to which exposure to neurotoxicants affects the neuroimmune response needs to be further studied.

Although microglial activation is mostly a beneficial response, it may change in chronic disease or in aging, losing their protective capacities or even developing neurotoxic properties^21,22^. In the same way, exposure to neurotoxicants may affect microglial cell function and contribute to pathophysiological changes in brain function. Due to the potential neurotoxicity of an inappropriate immune response, microglial activation is tightly regulated. Several inhibitory mechanisms involved in neuron-glia crosstalk participate in the regulation of microglial inflammatory responses, such as the CD200-CD200R1 interaction^23,24^. In the CNS, CD200 is a ligand mainly expressed in the cell membrane of neurons, but also of astrocytes and oligodendrocytes, while the inhibitory receptor CD200R1 is expressed in microglial cells. Alterations in the expression of CD200 and or CD200R1 in the CNS have been reported in neurological disorders^25–27^. The effect of neurotoxicants on CD200 or CD200R1 expression or function has not been evaluated so far.

We previously described that exposure of primary glial cell cultures to the parkinsonian neurotoxicants MPP+ and rotenone disrupted the immune response to a pro-inflammatory stimulus, LPS/IFN-γ, suggesting that changes in glial cell metabolism interfered with proper glial cell function^11^. The aim of the present work was to study whether MPP+ and rotenone interfered with the development of an anti-inflammatory response in glial cells. To this end, we treated mouse primary mixed glial and microglial cultures with IL4 in the absence and the presence of these neurotoxicants. We determined the effect of MPP+ and rotenone on IL4-induced expression of anti-inflammatory markers, the CD200-CD200R1 ligand-receptor pair, microglial phagocytosis and cell metabolism. Our results suggest that changes in glial cell metabolism are behind an impaired glial immune response induced by this type of agents, and may play a role in the neurodegeneration observed in Parkinson’s disease and other neurodegenerative disorders.

## Results

### Glial cell viability after MPP+ or rotenone exposure

In a previous study^11^, we performed concentration–response experiments to determine the working concentrations of MPP+ and rotenone that did not significantly affect the viability of glial cell cultures after a 24 h exposure. Thus, 10 and 25 μM MPP+ and 40 and 100 nM rotenone were used as the working concentrations in this study. No significant cell death was detected by propidium iodide staining in any of the experimental conditions assessed (see Supplementary Fig. 1).

### MPP+ and rotenone cause changes in the anti-inflammatory response induced by IL4 in primary glial cultures

The effect of MPP+ or rotenone treatment on the expression of anti-inflammatory genes in primary glial cell cultures was examined, as well as whether they had some effect on the development of the anti-inflammatory response induced by IL4. To this end, we determined the mRNA expression of the anti-inflammatory cytokines IL10, TGFβ and IL1ra, as well as of the molecules involved in tissue repair such as ARG1, MR, FIZZ1 and YM1, which are typical markers of an anti-inflammatory response.

**Figure 1:**
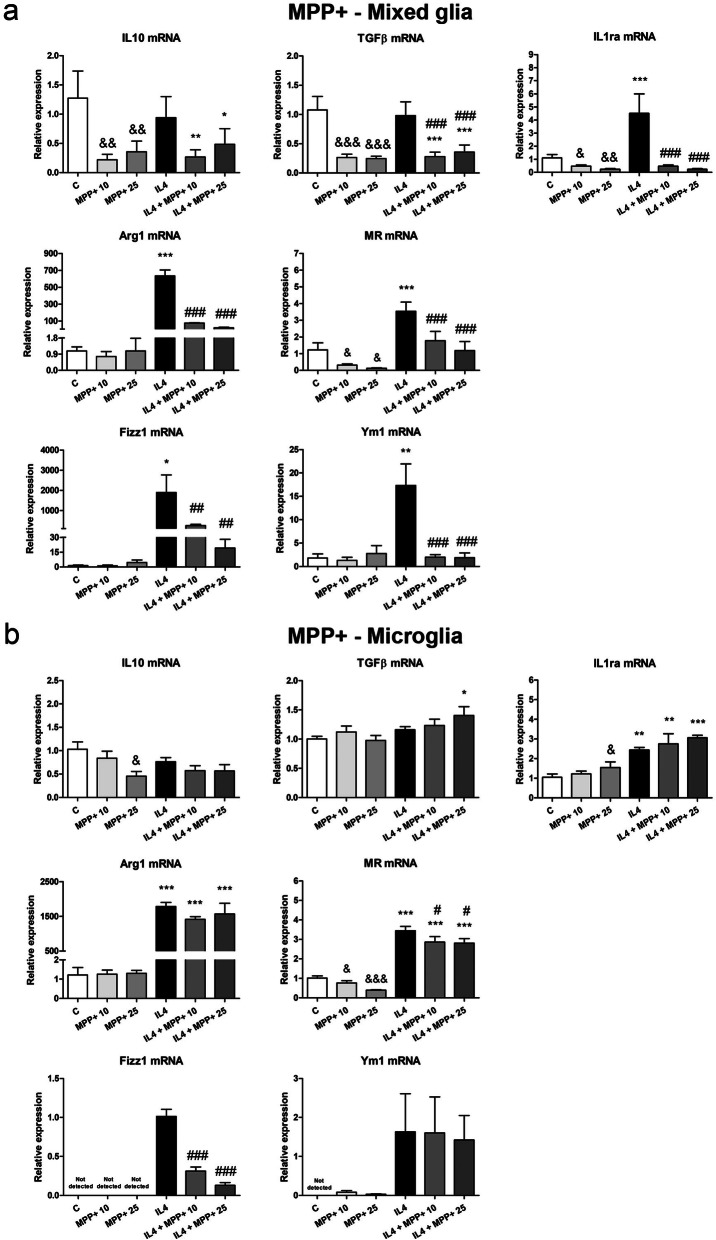
Effect of MPP+ treatment on the mRNA expression of anti-inflammatory markers. IL10, TGFβ, IL1ra, Arg1, MR, Fizz1 and Ym1 mRNA expression in primary (**a**) mixed glial cultures and (**b**) microglial cultures treated for 24 h with 10 or 25 μM MPP+, in the absence and presence of IL4 (50 ng/mL). Genes encoding Rn18s and β-actin were used as the reference genes. Bars correspond to the means + SEM of four independent experiments. *p < 0.05, **p < 0.01 and ***p < 0.001 vs control (C); ^#^p < 0.05, ^##^p < 0.01 and ^###^p < 0.001 vs IL4; one-way ANOVA and Newman-Keuls post-hoc test. ^&^p < 0.05, ^&&^p < 0.01 and ^&&&^p < 0.001 vs C, one-way ANOVA and Newman-Keuls post-hoc test considering only the IL4-free groups, which was performed to detect whether the high values observed in the IL4 group hindered the detection of a statistical significance for the effects of MPP+ alone.

**Figure 2:**
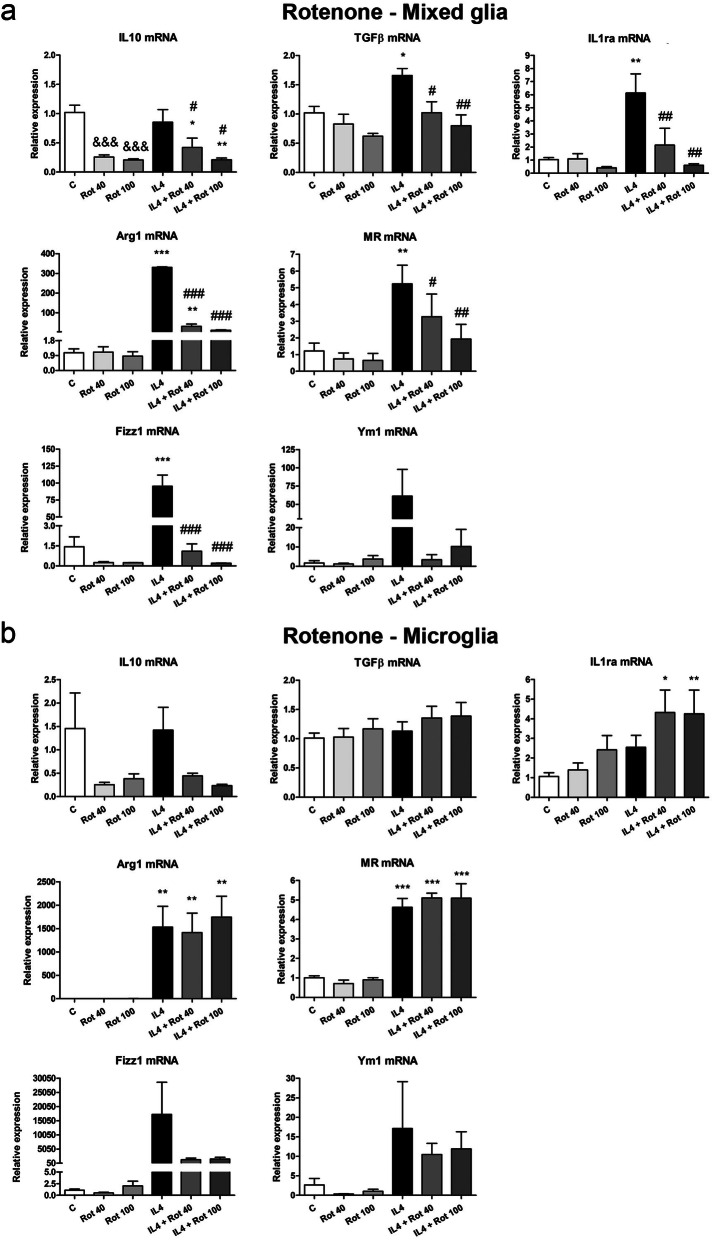
Effect of rotenone treatment on the mRNA expression of anti-inflammatory markers. IL10, TGFβ, IL1ra, Arg1, MR, Fizz1 and Ym1 mRNA expression in primary (**a**) mixed glial cultures and (**b**) microglial cultures treated for 24 h with 40 or 100 nM rotenone (Rot), in the absence and presence of IL4 (50 ng/mL). Genes encoding Rn18s and β-actin were used as the reference genes. Bars correspond to the means+ SEM of four independent experiments. *p < 0.05, **p < 0.01 and ***p < 0.001 vs control (C); ^#^p < 0.05, ^##^p < 0.01 and ^###^p < 0.001 vs IL4; one-way ANOVA and Newman-Keuls post-hoc test. ^&&&^p < 0.001 vs C, one-way ANOVA and Newman-Keuls post-hoc test considering only the IL4-free groups, which was performed to detect whether the high values observed in the IL4 group hindered the detection of a statistical significance of the effects of Rot alone.

In the mixed glial cultures, MPP+ treatment significantly decreased the expression of IL10, TGFβ, IL1ra and MR mRNAs (Fig. 1a), while rotenone significantly inhibited IL10 mRNA expression (Fig. 2a). MPP+ (Fig. 1b), but not rotenone (Fig. 1b), also decreased IL10 and MR mRNA levels in the microglial cultures. The expression of most of the anti-inflammatory markers assessed in this study significantly increased in IL4-treated mixed glial and microglial cultures (Figs. 1 and 2). However, MPP+ and rotenone clearly inhibited the IL4-induced mRNA expression of all the anti-inflammatory markers analyzed in the mixed glial cultures (Figs. 1a and 2a). MPP+ also inhibited the IL4-induced MR and Fizz1 mRNA expression in the microglial cultures (Fig. 1b). Both MPP+ and rotenone plus IL4 increased IL1ra mRNA expression in the microglial cultures (Figs. 1b and 2b).

**Figure 3:**
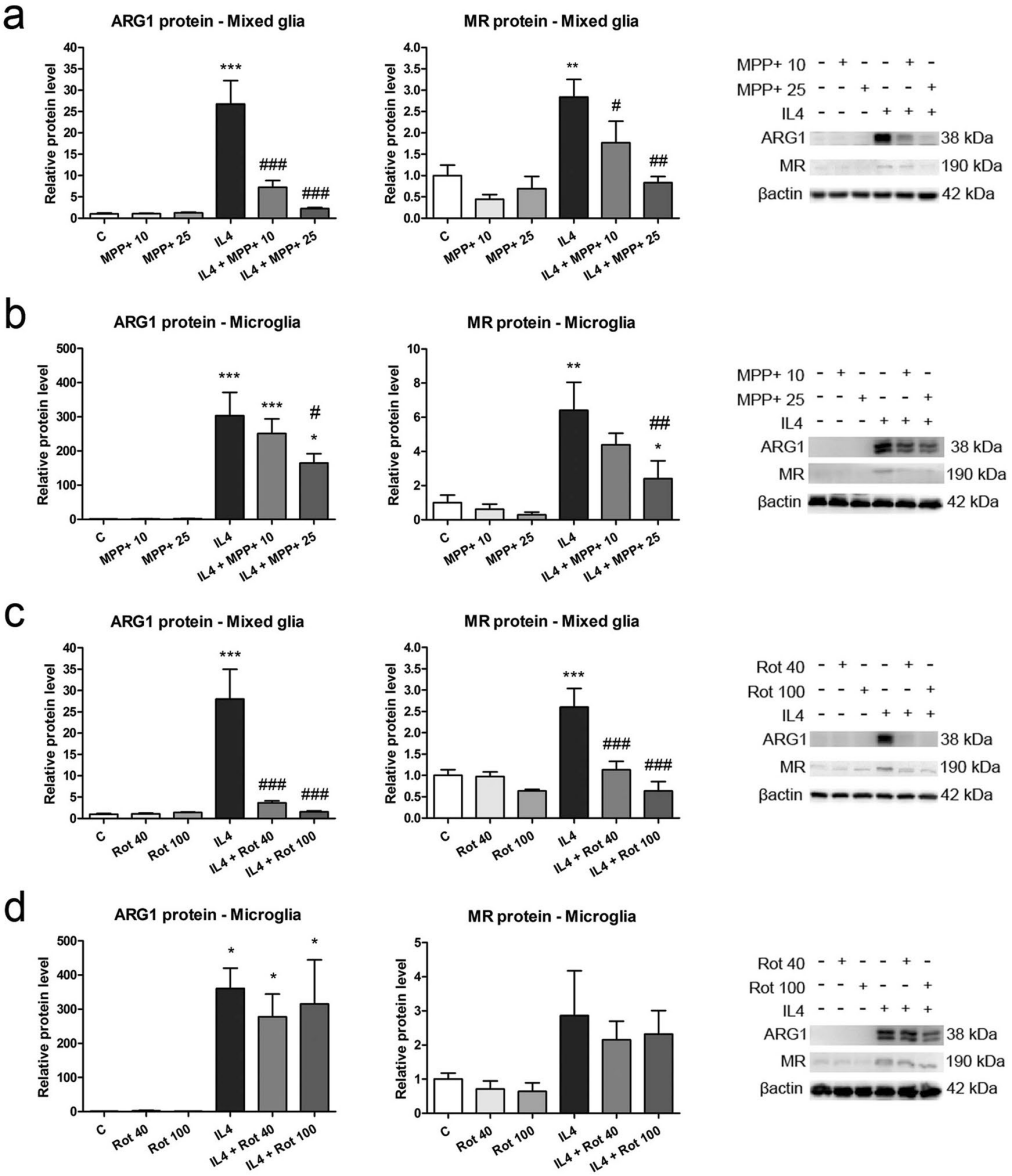
ARG1 and MR protein levels in primary glial cell cultures treated with MPP+ or rotenone. ARG1 and MR levels were determined in the total protein extracts from (**a**, **c**) mixed glial cultures and (**b**, **d**) microglial cultures treated with 10 or 25 μM MPP+ or 40 or 100 nM rotenone (Rot) for 24 h, in the absence or presence of IL4 (50 ng/mL). Images of representative western blots are presented. Bars correspond to the means+ SEM of four independent experiments. *p < 0.05, **p < 0.01 and ***p < 0.001 vs control (C); #p < 0.05, ^##^p < 0.01 and ^###^p < 0.001 vs IL4; one-way ANOVA and Newman-Keuls post-hoc test.

The changes observed in ARG1 and MR mRNA expression were further corroborated at the protein level. Protein levels of ARG1 and MR showed clear increases in the mixed glial and microglial cultures 24 h after IL4 treatment (Fig. 3a, d). These effects were inhibited by MPP+ in both types of cultures (Fig. 3a, b) and by rotenone in only the mixed glial cultures (Fig. 3c, d).

### Effect of MPP+ or rotenone on the CD200–CD200R1 system

The effect of MPP+ or rotenone on the expression of the CD200-CD200R1 system, one of the main inhibitory systems involved in regulating the microglial inflammatory response, was also evaluated. In the case of CD200, we considered the mRNA expression of two forms of the ligand, CD200full mRNA, which encodes the full-length form, and CD200tr mRNA, which encodes a truncated form that is able to interact with CD200R1 but does not activate the signal transduction pathway^28,29^.

**Figure 4:**
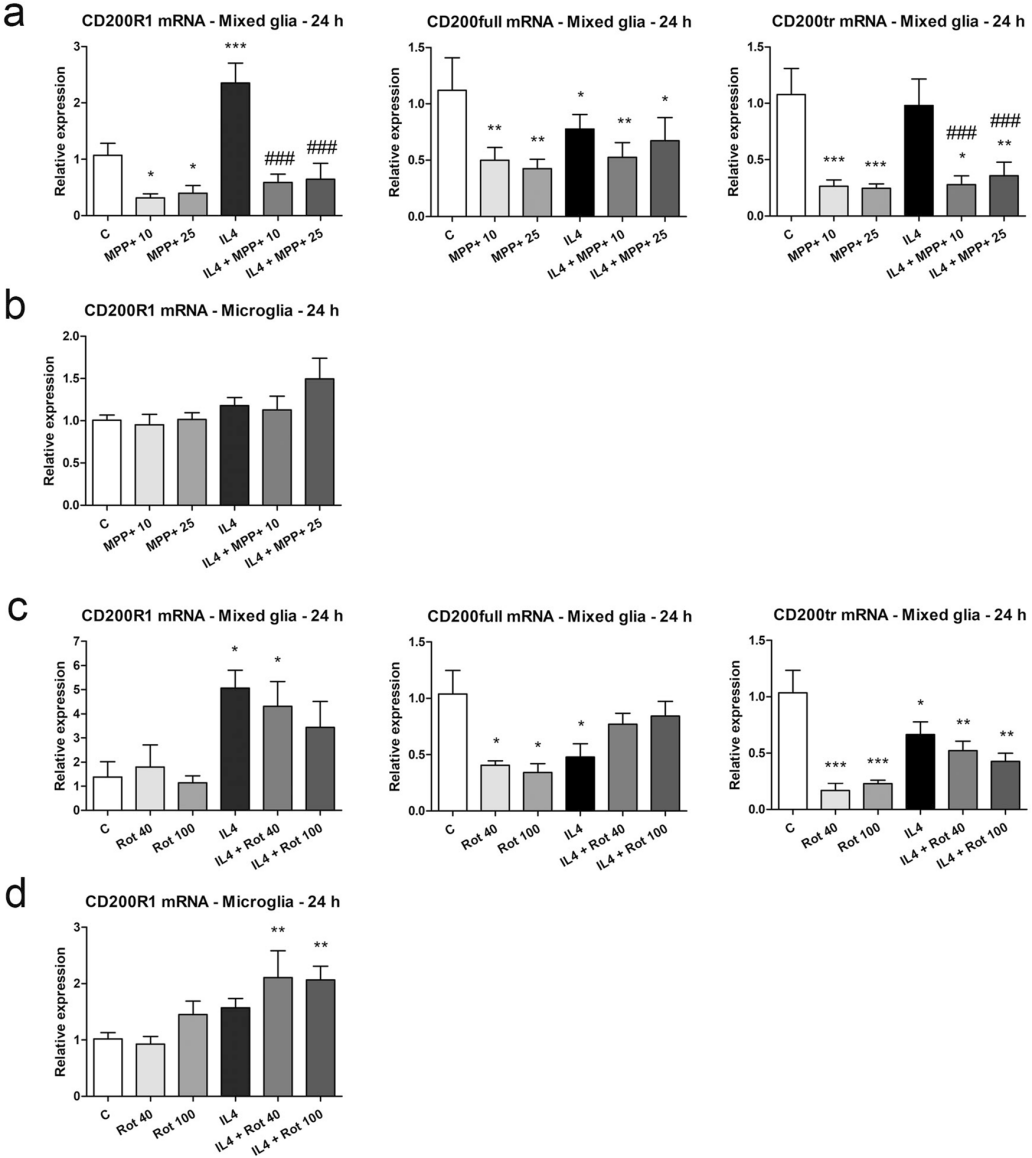
Effect of MPP+ or rotenone treatment on CD200 and CD200R1 mRNA expression. CD200full, CD200tr and CD200R1 mRNA expression in primary mixed glial cultures (**a**, **c**) and microglial cultures (**b**, **d**) treated for 24 h with 10 or 25 μM MPP+ (**a**, **b**) or 40 or 100 nM rotenone (Rot) (**c**, **d**), both in the absence and presence of IL4 (50 ng/mL). Genes encoding Rn18s and β-actin were used as the reference genes. Bars correspond to the means + SEM of four independent experiments. *p < 0.05, **p < 0.01 and ***p < 0.001 vs control (C); ###p < 0.001 vs IL4; one-way ANOVA and Newman-Keuls post-hoc test.

In the mixed glial cultures, MPP+ inhibited the mRNA expression of CD200R1 and CD200, both CD200full and CD200tr (Fig. 4a), while rotenone decreased the mRNA expression of CD200full and CD200tr (Fig. 4c). By contrast, neither MPP+ nor rotenone affected CD200R1 mRNA expression in the purified microglial cultures (Fig. 4b, d). IL4 significantly increased CD200R1 mRNA expression in the mixed glial cultures. Although this increase was also observed in the purified microglial cultures, it did not reach statistical significance. The IL4-induced increase in CD200R1 mRNA expression was strongly inhibited by MPP+ and to a lesser extent by rotenone. IL4 decreased CD200full and CD200tr mRNA expression in the mixed glial cultures, with MPP+ further inhibiting CD200tr mRNA expression.

### Microglial phagocytic activity is inhibited by MPP+, rotenone and IL4

**Figure 5:**
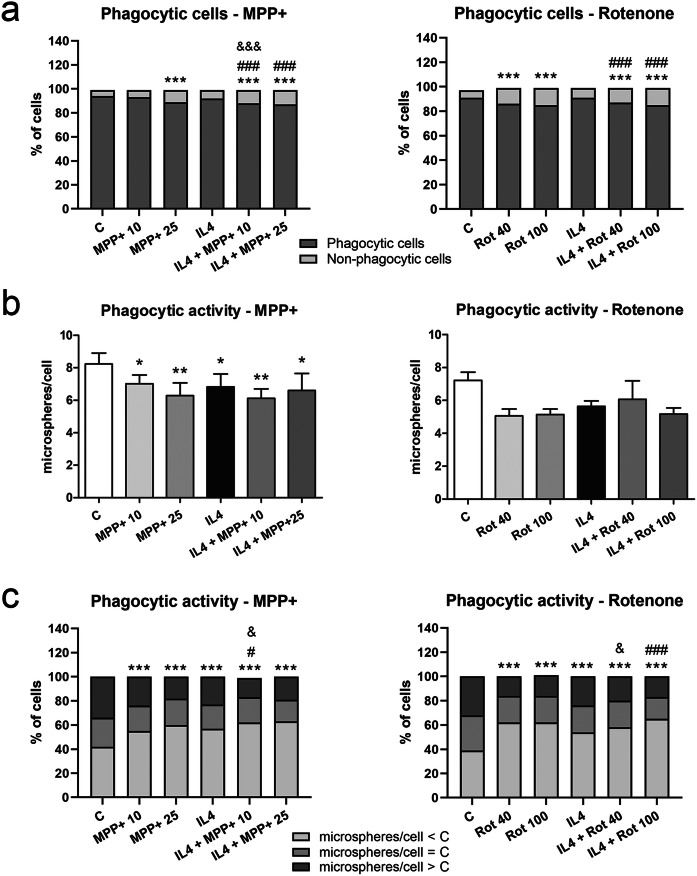
Phagocytosis in microglial cells exposed to MPP+ or rotenone. The phagocytosis of fluorescent microspheres was tested in primary microglial cultures treated with 10 or 25 μM MPP+ or 40 or 100 nM rotenone (Rot) in the absence and presence of IL4 (50 ng/mL). Intracellular microspheres were quantified after the immunofluorescence labeling of microglial cells using an anti-Iba1 antibody. (**a**) Classification of microglial cells into phagocytic (cells containing microspheres) or non-phagocytic (cells without microspheres) in the different experimental conditions. Bars correspond to percentages of the total amount of cells of 5 independent cultures for each experimental condition. p < 0.001 in the MPP+ and the rotenone experiments, Chi-square test; ***p < 0.001 vs C, ###p < 0.001 vs IL4, and ^&&&^p < 0.001 vs MPP+ or Rot alone; Chi-square tests. (**b**) Means of fluorescent microspheres per cell in phagocytic cells. Bars correspond to the means + SEM of 5 independent experiments. *p < 0.05 and **p < 0.01 vs. control (C); one-way ANOVA and Newman-Keuls post-hoc test. (**c**) Classification of phagocytic microglial cells into three groups by their degree of phagocytic activity: cells with fewer microspheres/cell than the mean value minus the variance in control cells, cells with microspheres/cell in the range of the mean value ± variance in control cells and cells with microspheres/cell higher than the mean value plus the variance in control cells. Bars correspond to percentages of the total amount of cells of 5 independent cultures for each experimental condition. p < 0.001 in the MPP+ and the rotenone experiments, Chi-square test; ***p < 0.001 vs C, ^#^p < 0.05 and ^###^p < 0.001 vs IL4, and ^&^p < 0.05 vs MPP+ or Rot alone; Chi-square tests.

In the presence of fluorescent latex microspheres, the phagocytosis in microglial cells was reduced by MPP+ , rotenone and IL4 (Fig. 5). The proportion of phagocytic cells was significantly dependent on the experimental situation, and a reduction was observed after MPP+ or rotenone treatment, both in the absence and the presence of IL4 (Fig. 5A). The average of microspheres/microglial cell was also significantly decreased by MPP+- and IL4, while a trend to decrease was observed in rotenone-treated microglia (Fig. 5B). We also observed that the proportion of phagocytic microglial cells showing low phagocytic activity (microspheres/cell lower than that of control) or high phagocytic activity (microspheres/cell higher than that of control) was significantly dependent on the experimental situation (Fig. 5C). The former was increased by MPP+ , rotenone and IL4, and a synergistic effect was observed in IL4-treated microglial cells in the presence of the neurotoxicants (Fig. 5C).

### MPP+ and rotenone inhibit metabolic activity and decrease intracellular ATP levels in mixed glia but not in microglial cultures

**Figure 6:**
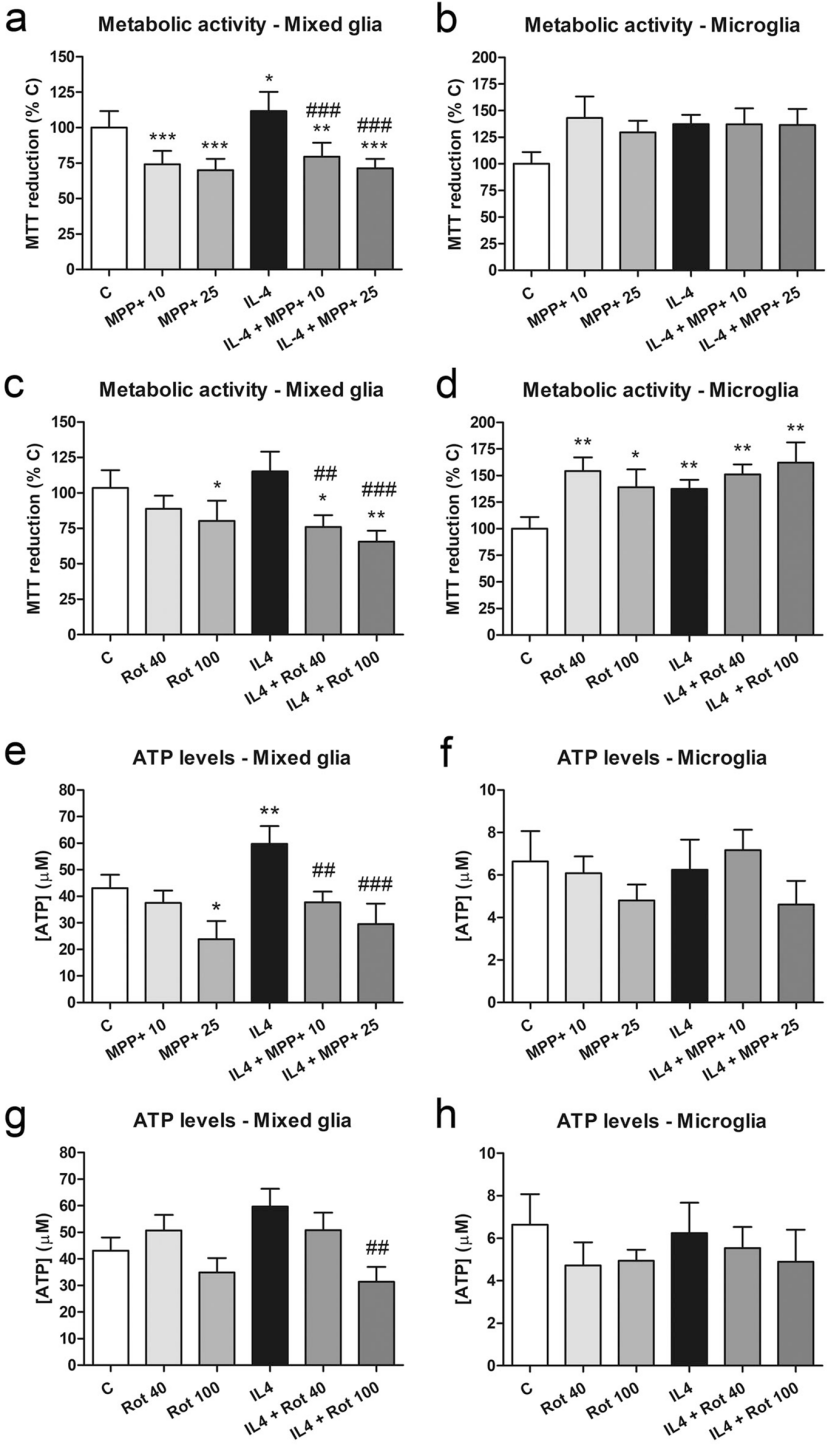
Metabolic activity and intracellular ATP levels in primary glial cell cultures treated with MPP+ or rotenone. (**a**–**d**) MTT reduction in mixed glial cultures (left column) and microglial cultures (right column) treated for 24 h with 10 or 25 μM MPP+ (**a**, **b**) or 40 or 100 nM Rot (**c**, **d**) in the absence and presence of IL4 (50 ng/mL). (**e**–**h**) Intracellular ATP levels in mixed glial cultures (left column) and microglial cultures (right column) treated with 10 or 25 μM MPP+ (**e**, **f**) or 40 or 100 nM rotenone (Rot) (**g**, **h**) for 24 h, in the absence and presence of IL4 (50 ng/mL). Bars correspond to the means + SEM of five independent experiments. *p < 0.05, **p < 0.01 and ***p < 0.001 vs control (C); ^##^p < 0.01 and ^###^p < 0.001 vs IL4; one-way ANOVA and Newman-Keuls post-hoc test.

We used the MTT reduction assay as an indirect measurement of the metabolic activity of the glial cell cultures. Mixed glial cell cultures treated with 10 or 25 µM MPP+ showed a significant decrease in MTT reduction 24 h after treatment (Fig. 6a). IL4 treatment resulted in an increase in MTT reduction, which was abrogated by MPP+. Similar results were obtained with rotenone in the mixed glial cultures (Fig. 6c). By contrast, an increased MTT reduction was observed in microglial cultures treated with MPP+ (Fig. 6b) or rotenone (Fig. 6d), with the effect in the rotenone-treated cultures being significant.

As MPP+ and rotenone are inhibitors of the mitochondrial respiratory chain, we determined whether intracellular ATP levels were modified in glial cells under our experimental conditions. In general, ATP levels tended to decrease in MPP+- and rotenone-treated cultures (Fig. 6e–h), with this decrease being statistically significant in the mixed glial cells treated with the highest concentration of MPP+ (Fig. 6e). By contrast, ATP levels were increased in IL4-treated mixed glial cell cultures (Fig. 6e, g). However, this increase was significantly abrogated by 10 and 25 μM MPP+ and 100 nM rotenone (Fig. 6e, g). ATP levels were not significantly affected in the microglial cultures, although ATP levels tended to decrease in response to MPP+ or rotenone exposure (Fig. 6f, h).

### Metabolic reprogramming in IL4-treated glial cultures: effect of MPP+ and rotenone

Macrophages/microglial cells developing a pro-inflammatory phenotype switch to glycolysis to obtain energy, during which lactate production from pyruvate and the pentose phosphate pathway are potentiated. By contrast, macrophages/microglial cells developing an anti-inflammatory phenotype show increased oxidative phosphorylation^30, 31^. However, MPP+ and rotenone inhibition of complex I of the mitochondrial respiratory chain may interfere with this metabolic reprogramming.

To explore how glial cells may adapt their metabolism to be able to respond to IL4 in the presence of MPP+ or rotenone, the mRNA expression of the genes encoding proteins that are critical for the glycolytic and oxidative pathways was determined. We focused on molecules (a) critical for reprogramming during M1 and M2 polarization, such as PPARγ coactivator-1β (PGC1β) and carbohydrate kinase-like protein (CARKL), and (b) playing a central role in glycolysis, such as the glucose transporter GLUT1, the glycolytic enzyme phosphofructokinase (PFKP) and the glycolysis activator HIF1α.

**Figure 7:**
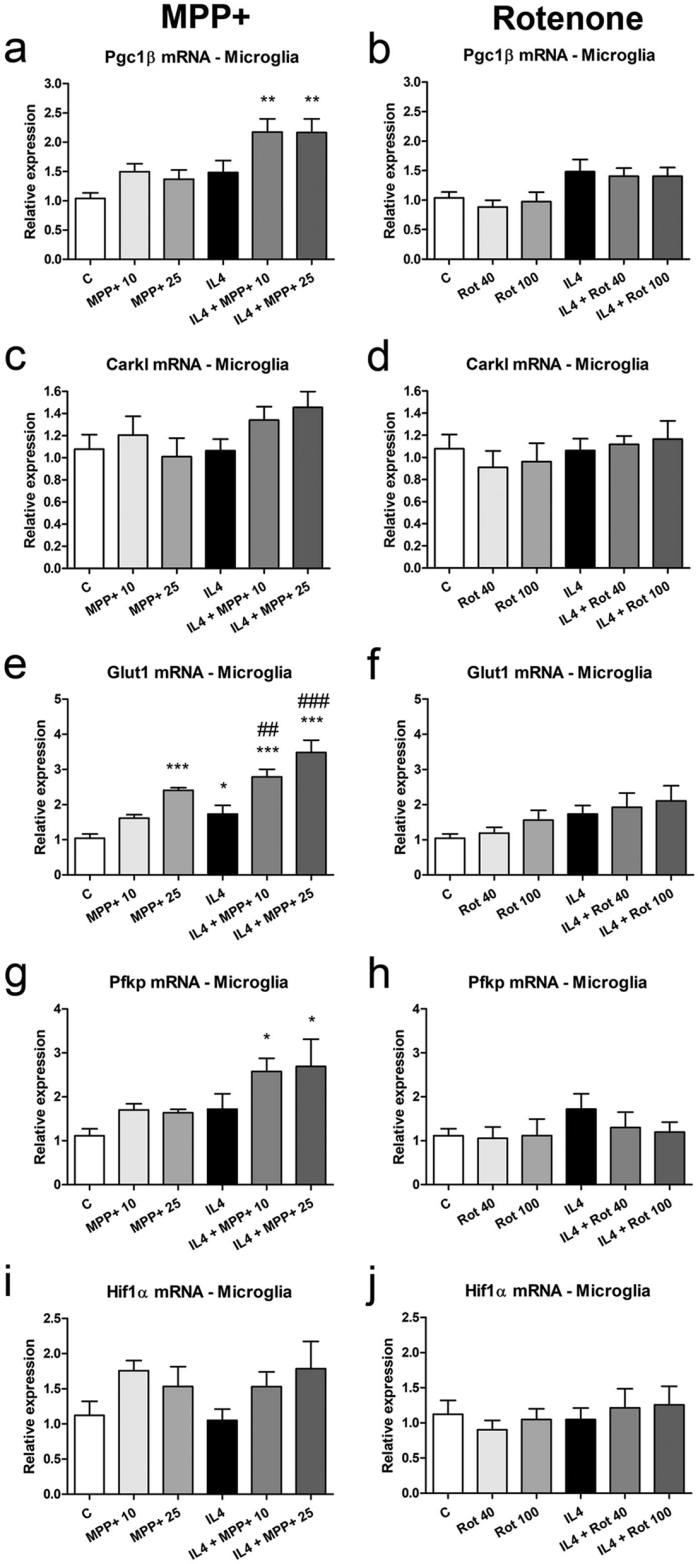
Effect of MPP+ or rotenone treatment on the expression of genes involved in regulating energy metabolism in microglial cultures. (**a, b**) Pgc1β, (**c**, **d**) Carkl, (**e**, **f**) glucose transporter (Glut)1, (**g**, **h**) Pfkp and (**i**, **j)** Hif1α mRNA expression in microglial cultures treated for 24 h with 10 or 25 μM MPP+ (left column) or 40 or 100 nM rotenone (Rot, right column), in the absence and presence of IL4 (50 ng/mL). Genes encoding Rn18s and β-actin were used as the reference genes. Bars correspond to the means + SEM of four independent experiments. *p < 0.05, **p < 0.01 and ***p < 0.001 vs control (C); ##p < 0.01 and ###p < 0.001 vs IL4; one-way ANOVA and Newman-Keuls post-hoc test.

Pgc1β mRNA levels were significantly increased in IL4-treated microglial cultures in the presence of MPP+. They were also elevated in IL4-treated microglial cultures, both in the absence and presence of rotenone (Fig. 7a, b). Carkl mRNA levels were not modified in our experimental conditions (Fig. 7c, d). Regarding the expression of the molecules involved in glycolysis, Glut1 mRNA expression was increased in the microglial cells treated with MPP+ or IL4, showing a further increase in cells treated with both IL4 and MPP+ (Fig. 7e, f). Increased Pfkp mRNA levels were also observed in cultures treated with both IL4 and MPP+ (Fig. 7g, h). Microglial cells treated with MPP+ tended to increase Hif1α mRNA levels (Fig. 7i, j).

**Figure 8:**
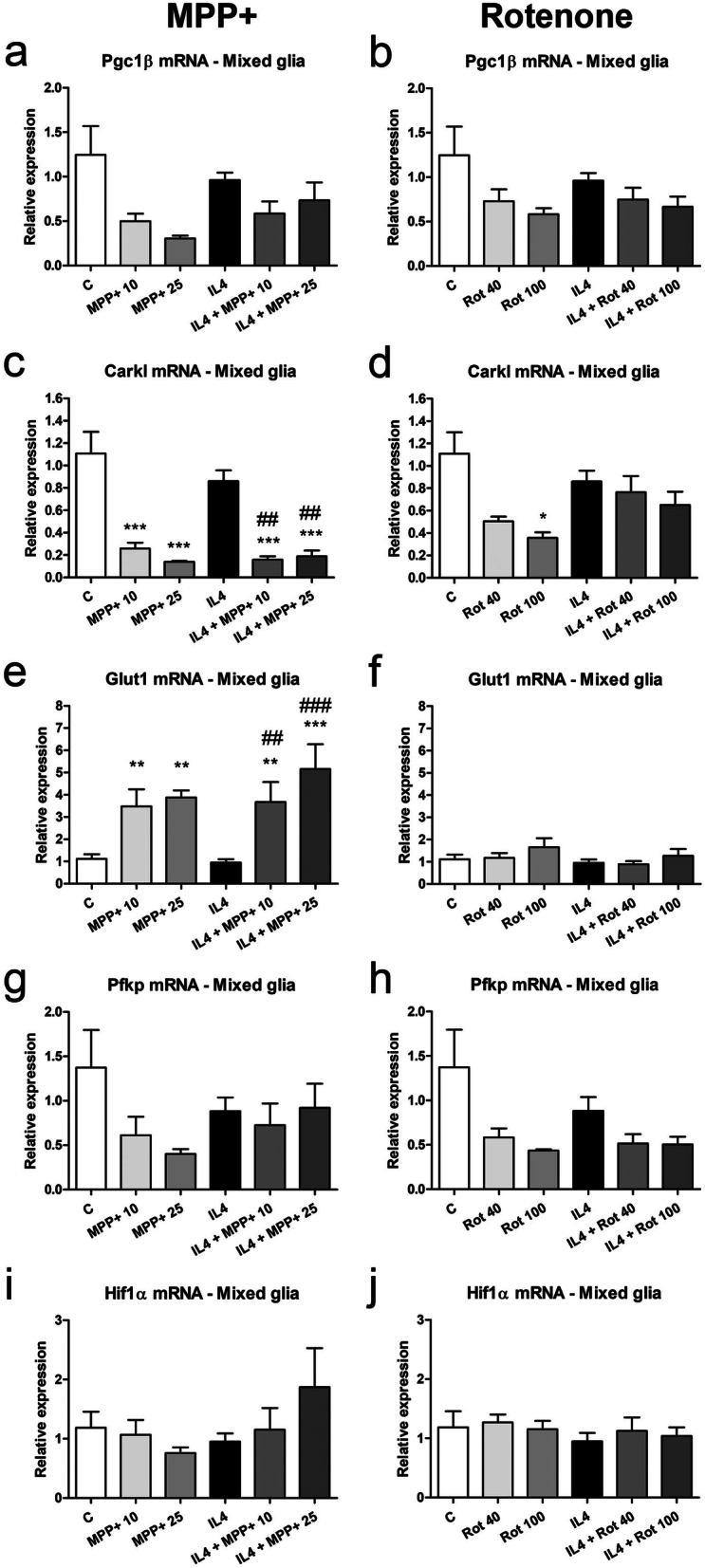
Effect of MPP+ or rotenone treatment on the expression of genes involved in regulating energy metabolism in mixed glial cultures. (**a**, **b**) Pgc1β, (**c**, **d**) Carkl, (**e**, **f**) the glucose transporter (Glut)1, (**g**, **h**) Pfkp and (**i**, **j**) Hif1α mRNA expression in mixed glial cultures treated for 24 h with 10 or 25 μM MPP+ (left column) or 40 or 100 nM rotenone (Rot, right column), in the absence and presence of IL4 (50 ng/mL). Genes encoding Rn18s and β-actin were used as the reference genes. Bars correspond to the means + SEM of four independent experiments. *p < 0.05, **p < 0.01 and ***p < 0.001 vs control (C); ^##^p < 0.01 and ^###^p < 0.001 vs IL4; one-way ANOVA and Newman-Keuls post-hoc test.

Changes in the expression of these metabolic enzymes were much more apparent in the mixed glial cultures (Fig. 8), mainly after MPP+ treatment, and differed from those observed in microglial cultures. Thus, Pgc1β (Fig. 8a, b) and Carkl mRNA levels (Fig. 8c, d) significantly decreased in MPP+ -treated mixed glial cultures, both in the absence and presence of IL4. Carkl mRNA expression was also inhibited by rotenone in IL4-treated mixed glial cultures (Fig. 8c, d). MPP+ increased Glut1 mRNA expression in the mixed glial cultures (Fig. 8e, f), which was potentiated by IL4. Pfkp mRNA levels tended to decrease in all our experimental conditions (Fig. 8g, h). No changes were detected in Hif1α mRNA levels (Fig. 8i, j).

**Figure 9:**
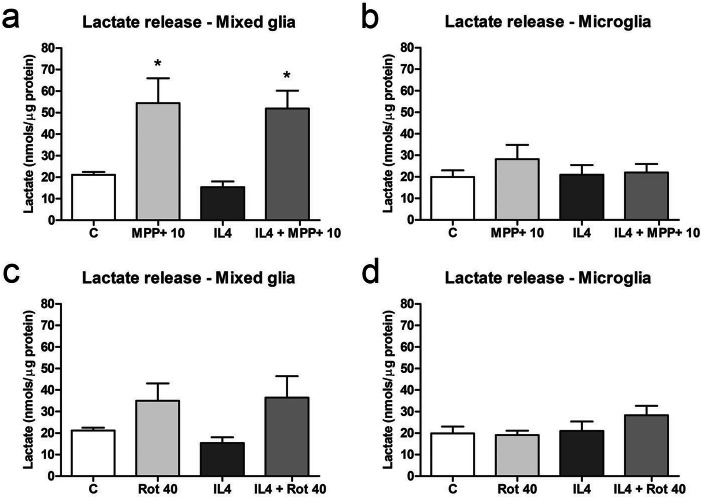
Lactate release in primary glial cell cultures treated with MPP+ or rotenone. Extracellular lactate levels in mixed glial cultures (left column) and microglial cultures (right column) treated with 10 μM MPP+ (**a**, **b**) or 40 nM rotenone (Rot) (**c**, **d**) for 24 h, in the absence and presence of IL4 (50 ng/mL). Bars correspond to the means + SEM of three independent experiments. *p < 0.05 vs control (C); one-way ANOVA and Newman-Keuls post-hoc test.

To further explore metabolic reprogramming in glial cell cultures exposed to MPP+ or rotenone and IL4, lactate production, the end product of aerobic glycolysis, was measured as an index of the glycolytic flux. In mixed glial cultures, extracellular lactate levels significantly increased after MPP+ -treatment, both in the absence and presence of IL4, while the levels tended to increase after rotenone treatment (Fig. 9a, c). No changes in the extracellular lactate levels were observed in microglial cultures (Fig. 9b, d).

## Discussion

In the present study, we show that the expression of anti-inflammatory molecules is impaired in glial cells exposed to the neurotoxic agents MPP+ and rotenone. The basal expression levels of anti-inflammatory molecules were decreased in primary mixed glial and microglial cultures after MPP+ or rotenone treatment. Furthermore, these neurotoxicants also impaired the development of an anti-inflammatory phenotype in IL4-treated glial cultures. MPP+- and rotenone-induced changes in the metabolic activity of glial cells may contribute to the impairment of the immune responses observed.

The neurotoxic effect of the parkinsonian neurotoxicants MPP+ and rotenone (as well as other molecules that inhibit the respiratory electron transport chain) has been widely reported in studies using in vivo and in vitro experimental approaches. Dopaminergic cells are especially sensitive to the toxic effects of these compounds, with the mechanism of action of the neurotoxicants in neuronal cells being widely studied. However, there is less information on their effects on glial cells^4^. In a recent study^11^, we reported that MPP+ and rotenone, in particular, disrupted the development of immune responses in glial cells exposed to a pro-inflammatory stimulus (LPS/IFN-γ).

In the present work, we showed that the response of glial cells to an anti-inflammatory stimulus (IL4) was also impaired by MPP+ and rotenone. Both the basal expression levels of anti-inflammatory molecules and the IL4-induced expression of anti-inflammatory markers were significantly reduced by MPP+ and rotenone in glial cultures. These effects were more pronounced in the mixed glial cultures than in the microglial cultures. Ferger et al.^32^ reported that rotenone had no effect on LPS-induced pro-inflammatory cytokine production in primary microglial cells, but that it did inhibit IL4-induced arginase activity and expression. The authors also demonstrated that rotenone inhibited the IL4-induced reduction of LPS-stimulated cytokine secretion. In the present study, we did not detect changes in IL4-induced ARG1 mRNA expression in microglial cultures exposed to MPP+ or rotenone, although IL4-induced ARG1 protein expression was inhibited in microglial cultures treated with MPP+. However, we did observe a marked reduction in the expression of ARG1 and other anti-inflammatory markers in IL4-treated mixed glial cultures exposed to the neurotoxicants. The differences between our results and those of Ferger et al.^32^ could be due to the lower concentration of IL4 (10 ng/mL) and/or the higher concentration of rotenone (200 nM) used by these authors.

The inhibition of the response of glial cells to IL4 may have special relevance in those situations where glial cells produce IL4 as a compensative response to counteract alterations in neuronal function or to participate in the resolution of an inflammatory response. In this sense, Hünher et al.^33^ show that endogenous IL4 production in neuron-glia mesencephalic cultures is neuroprotective against MPP+ neurotoxicity, and suggest that this effect is mediated by factors released by glial cells in response to IL4.

In addition, MPP+ and rotenone impaired the phagocytosis in microglial cells. The proportion of phagocytic cells was reduced by MPP+ and rotenone exposure. Moreover, the proportion of the phagocytic cells showing lower phagocytic activity than control was increased by MPP+, rotenone and IL4, effect that was enhanced in the IL4-treated cultures exposed to the neurotoxicants. Chang et al.^34^ and Zhang et al.^35^ showed increased phagocytosis in BV2 cells treated with rotenone, at a concentration lower than the one used in the present study. A different sensitivity of this murine cell line to the effect of rotenone could account for the differences observed.

In order to study possible mechanisms involved in the MPP+- and rotenone-induced changes in the inflammatory response and the phagocytic activity of glial cells, we looked at the ligand-receptor pair CD200-CD200R1, which has a neuroimmune regulatory function. The microglial receptor CD200R1 is involved in the inhibition of the pro-inflammatory phenotype in microglial cells. Its ligand, CD200, is mainly expressed by neurons, but also by astroglial cells^24^. Several studies have shown that CD200R1 stimulation inhibits the expression of pro-inflammatory cytokines and induces the expression of the anti-inflammatory markers IL10 and ARG1 in glial cells treated with pro-inflammatory stimuli^36–39^. In addition, Yi et al.^39^ reported that CD200R1 is necessary for the induction of an anti-inflammatory phenotype by IL4 in microglial cells. A role for CD200-CD200R1 interaction in the control of phagocytosis in primary microglia^40^ and macrophages^41^ has also been suggested.

In previous studies, we showed that CD200R1 expression is decreased in response to pro-inflammatory stimuli (LPS or LPS/IFNγ)^42,43^, suggesting that the inhibitory function of CD200R1 is abrogated and the pro-inflammatory phenotype potentiated. In this study, we observed that CD200R1 mRNA levels were increased in IL4-treated mixed glial cultures, indicating that the inhibitory function of CD200R1 is enhanced to promote an anti-inflammatory phenotype. On the contrary, CD200 mRNA expression was inhibited by IL4 in astrocytes. As IL4 has been demonstrated to increase CD200 expression in neurons^44^ and since CD200 expression has been reported to be decreased in neuronal cultures from IL4−/− mice^45^, our results suggest that IL4 regulates CD200 differently in neurons and astrocytes. MPP+ inhibited both the basal and IL4-induced expression of CD200R1 mRNA in mixed glial cultures, while rotenone inhibited IL4-induced CD200R1 mRNA expression. MPP+ and rotenone also inhibited CD200full and CD200tr mRNA expression both in the absence and presence of IL4. These results indicate that the neurotoxicants impair the CD200-CD200R1 system. As CD200R1 appears to participate in the induction of IL4-induced anti-inflammatory phenotype in microglial cells^39^, these alterations may contribute to the reduced expression of anti-inflammatory markers observed after MPP+ or rotenone treatment both in the absence and in the presence of IL4. On the contrary, CD200R1 mRNA expression was increased in IL4-treated microglial cultures exposed to MPP+ or rotenone, which showed few changes in the expression of anti-inflammatory markers.

As MPP+ and rotenone are inhibitors of the respiratory electron transport chain, the metabolic activity and the ATP production in glial cells may be compromised by these neurotoxicants^46,47^, which may contribute to the inhibited immune function observed. Significant changes in intracellular ATP levels were observed in the mixed glial cultures treated with MPP+ or rotenone: decreased ATP levels were observed for 25 µM MPP+, while 10 and 25 µM MPP+ inhibited IL4-increased ATP levels. ATP levels were also decreased in the mixed glial cultures treated with IL4 and 100 nM rotenone. These results suggest that IL4-treated mixed glial cultures do not meet the metabolic demand needed to develop an anti-inflammatory phenotype in the presence of the neurotoxicants. There were no significant changes in the microglial cultures in any of our experimental conditions, although ATP levels tended to decrease after MPP+ or rotenone treatment. In addition, the response of microglial cultures to IL4 in the presence of the neurotoxicants was not as affected as that of mixed glial cultures. Thus, microglial cells appear to be more resistant to the toxic effects of MPP+ and rotenone than mixed glial cultures. Curiously, the MTT assay results showed that metabolic activity was reduced in the mixed glial cultures, but increased in the microglial cultures exposed to MPP+ or rotenone, suggesting that microglial cells activate alternative but less productive ATP-generating pathways or have a higher rate of ATP consumption in response to the presence of the neurotoxicants.

It is suggested that metabolic reprogramming is behind the control of immune cell activation, although immunometabolism is still an emerging field in the context of glial cells^48,49^. In macrophages, specific patterns of metabolic activity have been associated with different phenotypes with particular energy requirements^30,31^. A M1 pro-inflammatory phenotype involved in pathogen elimination requires a rapid and intense response, which would need increased aerobic glycolysis to meet the energy demands. On the contrary, a M2 anti-inflammatory and repairing phenotype requires a maintained and long-lasting energy supply, mainly from oxidative phosphorylation. Although still underexplored, metabolic shifts have also been described in activated microglial cells^50–53^; however, some differences are thought to exist between microglia and peripheral macrophages. Thus, while studies on microglial cells have reported increased glycolysis after a pro-inflammatory stimulus similar to that observed in macrophages, no increased oxidative phosphorylation has been observed in microglial cells after an anti-inflammatory stimulus^43^. Indeed, Durafourt et al.^54^, comparing the polarization of human microglia and macrophages, suggested that microglial cells are more reluctant to adopt an M2 phenotype than macrophages. Astroglial cells are mainly glycolytic, but increase glucose utilization and become more oxidative in response to pro-inflammatory stimuli^55^, while decreasing glucose utilization in response to anti-inflammatory stimuli^56^.

In order to evaluate the metabolic reprogramming in glial cells exposed to MPP+ or rotenone and IL4, we evaluated the expression of key enzymes and molecules associated with glycolysis and oxidative metabolism, as well as lactate release as an index of aerobic glycolysis.

Vats et al.^57^ demonstrated that PGC1β, a regulator of the beta-oxidation of fatty acids and oxidative phosphorylation, is critical for M2 macrophage polarization, inducing fatty acid oxidation and mitochondrial biogenesis and inhibiting pro-inflammatory cytokine production in IL4-treated macrophages. In microglial cultures, Pgc1β mRNA levels tended to increase in response to MPP+ or IL4 alone. This tendency was also observed in the IL4-treated cultures exposed to rotenone, but a significant increase was observed in the IL4-treated cultures exposed to MPP+. These results suggest that although MPP+ and rotenone inhibit oxidative phosphorylation, microglial cells treated with both MPP+ and IL4 may increase their PGC1β expression to increase oxidative metabolism as a compensatory response. On the contrary, a trend to decrease Pgc1β mRNA levels was observed in mixed glial cultures.

In macrophages, CARKL, which is involved in regulating the pentose phosphate pathway, is required for metabolic reprogramming during M1 and M2 polarization^58^. CARKL catalyzes the production of sedoheptulose-7-phosphate, and its downregulation leads to the stimulation of the pentose phosphate pathway and vice versa. CARKL expression in macrophages is rapidly downregulated in response to LPS stimulation and slightly increased in response to IL4 treatment. CARKL downregulation appears to be critical for M1 polarization, given that CARKL activation results in the repression of the expression of pro-inflammatory genes. In microglial cultures, Carkl mRNA expression was not affected in our experimental conditions, suggesting that the glycolytic flux into the pentose phosphate pathway was maintained at basal levels. By contrast, previous results of our group showed a strong inhibition of Carkl mRNA in microglial cells treated with LPS/IFNγ^11^. In agreement with these results, Orihuela et al.^51^ reported that although macrophages and microglia increase glycolysis in response to pro-inflammatory stimuli, macrophages increase oxidative phosphorylation in response to IL4, but microglial cells have an oxidative metabolic state that is similar to that of non-stimulated cells following IL4 treatment. Nevertheless, MPP+ significantly decreased Carkl mRNA expression in both the absence and the presence of IL4 in the mixed glial cultures, and reduced Carkl mRNA expression was also observed after rotenone treatment alone.

IL4 stimulation of microglial cells elicits decreased glucose consumption and lactate production^48^. However, we observed significantly increased Glut1 mRNA levels in IL4-treated microglial cultures. MPP+ also increased Glut1 mRNA expression, further increasing the IL4-induced rise in expression. Glut1 mRNA expression levels also tended to increase after rotenone treatment. These increases suggest that an additional uptake of glucose is promoted to compensate for the reduced oxidative metabolism elicited by the parkinsonian neurotoxicants. In agreement with this, the mRNA expression of PFKP, which is one of the most important regulatory enzymes of glycolysis, was also increased in IL4-treated cultures exposed to MPP+. However, the expression of HIF1α, which potentiates glycolysis^59,60^, only showed a trend to increase in MPP+ -treated microglial cultures. In the mixed glial cultures, MPP+ significantly increased Glu1 mRNA expression.

Altogether, these results suggest that IL4-stimulated microglial cells exposed to MPP+ or rotenone tended to increase both oxidative metabolism and the glycolytic flux to compensate for the inhibition of oxidative phosphorylation by the complex I inhibitors. Curiously, no increase in lactate release was observed in MPP+- or rotenone- treated microglial cultures. However, the results obtained in the mixed glial cultures suggest that glycolysis and the pentose phosphate pathway, but not oxidative phosphorylation, are enhanced in mixed glial cultures. The increased extracellular lactate levels observed in mixed glial cultures treated with MPP+ and rotenone would corroborate this hypothesis.

**Figure 10:**
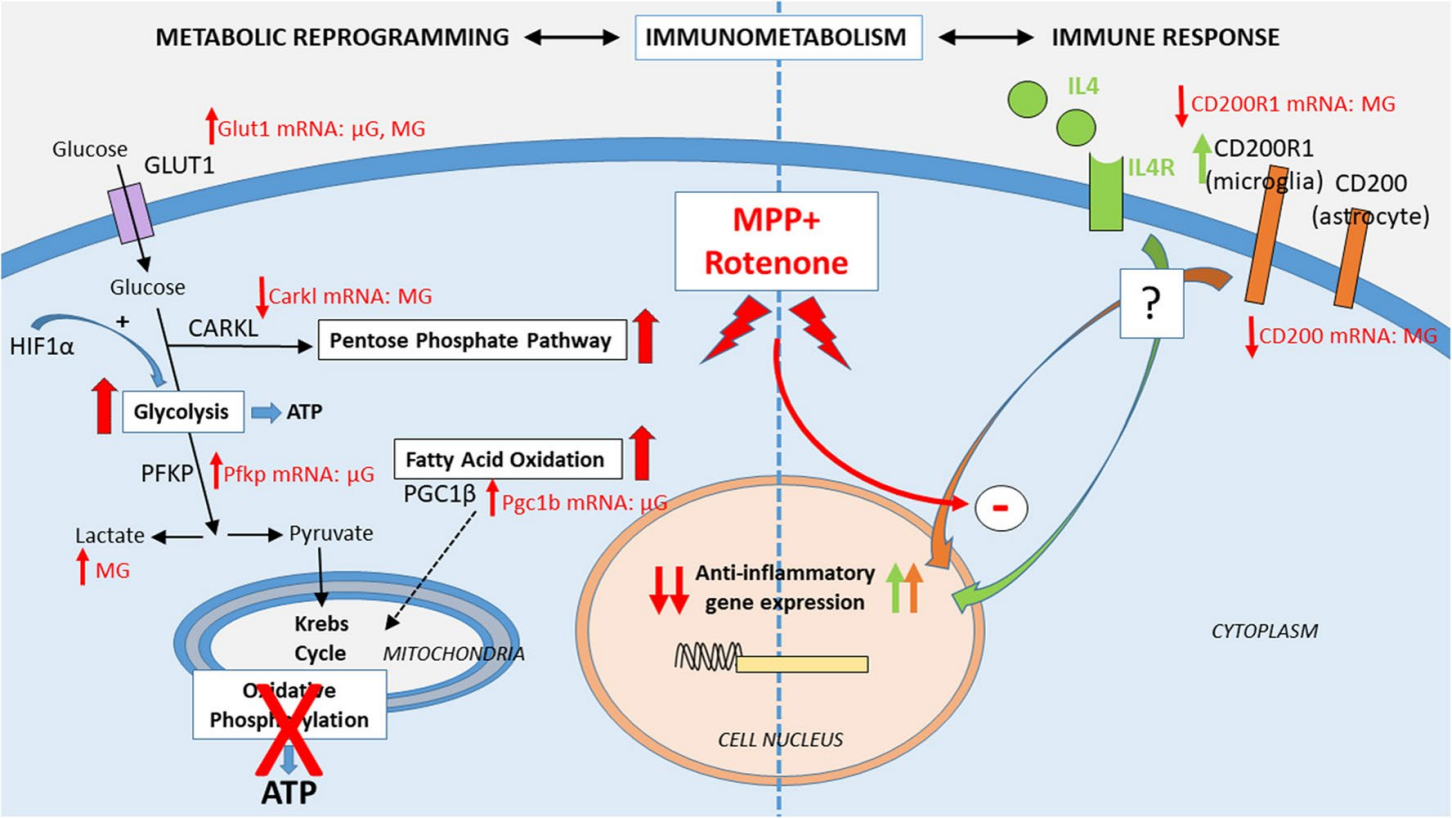
Summary of the effects of MPP+ and rotenone exposure on IL4-induced immune response on primary glial cell cultures. Immunometabolism refers to the close relationship existing between immune response and cell metabolism and the hypothesis that metabolic reprogramming is behind the development of a proper immune response by glial cells. IL4 treatment induces the expression of anti-inflammatory markers and CD200R1, and the stimulation of the CD200R1 immune receptor in microglial cells may play a role in the former by a still underexplored mechanism (right side of the figure, green and orange arrows). MPP+ and rotenone are inhibitors of the mitochondrial respiratory chain and consequently impair oxidative phosphorylation, which is associated to changes in the expression of genes encoding proteins involved in the control of glial cell metabolism in both microglial (µG) and mixed glial (MG) cultures (left side of the figure, red arrows). The inhibition of oxidative phosphorylation by the neurotoxicants stimulates aerobic glycolysis (associated to lactate production) and the pentose phosphate pathway in mixed glial cultures, while fatty acid oxidation and not the pentose phosphate pathway is stimulated in microglial cell cultures. This impairment may be responsible for the inhibition of the expression of anti-inflammatory markers observed in mixed glial cultures and, to a lesser extent, in microglial cultures after MPP+ and rotenone treatment. *CARKL* sedoheptulokinase; *GLUT1* glucose transporter 1; *HIF1α* hypoxia-inducible factor 1, alpha subunit; *PFKP* phosphofructokinase, platelet; *PGC1β* peroxisome proliferator-activated receptor gamma coactivator 1, beta.

In summary, our results show that MPP+ and rotenone disrupt the expression of anti-inflammatory markers in glial cells, probably by affecting glial cell metabolism and impairing metabolic reprogramming associated with glial activation (Fig. 10). These results support our previous observation that the immune responses of glial cells are compromised by neurotoxicants that inhibit the mitochondrial electron transport chain, which can affect the development of both the pro- and the anti-inflammatory phenotype. Apparently, microglial cultures are more resistant to the action of the neurotoxicants than mixed glial cultures, which contain 25% of microglia and 75% of astrocytes. A greater sensitivity of astrocytes to the effect of the neurotoxicants and subsequent alterations in astrocyte-microglia crosstalk may contribute to this observation. In fact, MPP+ and rotenone induced changes in the CD200-CD200R1 system, which is one of the mechanisms involved in astrocyte-microglia crosstalk. However, further studies on the effect of MPP+ and rotenone on astrocyte-enriched cultures are needed to corroborate this hypothesis. Molecules that inhibit oxidative phosphorylation may contribute to the development of Parkinson’s disease not only through their direct toxic effects on dopaminergic neurons, but also through their direct effects on glial cells, which may involve the loss of the metabolic reprogramming required to cope with immune challenges. Interestingly, it has been recently suggested that microglia metabolism may be an additional target to modulate microglial activated phenotype through metabolic reprogramming in neurodegenerative disorders^48,53,61^.

## Methods

Experiments were carried out in accordance with European Union directives (2010/63/EU) and Spanish regulations (Real Decreto 1386/2018) on the use of laboratory animals. They were approved by the Ethics and Scientific Committee of the University of Barcelona and CSIC (reference number OB-404/17).

### Cell cultures

Primary mixed glial cultures were prepared from the cerebral cortex of 1 to 3-days-old male and female C57Bl/6 mice, as previously described^62^. Cells were cultured in Dulbecco’s Modified Eagle Medium-F12 Nutrient Mixture (Gibco, Thermo Fisher Scientific, Waltham, MA, USA), that was supplemented with 10% heat-inactivated fetal bovine serum (Gibco), 20 U/mL penicillin-20 μg/mL streptomycin (Gibco), and 0.5 μg/mL amphotericin B (Fungizone; Gibco). The medium was replaced once a week. The cultures were used at 21 DIV. Primary microglia-enriched cultures were obtained from mixed glial cultures at 21 DIV, using the mild trypsinization method previously described by our group ^63^. Microglia enriched cultures were used 24 h after isolation.

### Cell culture treatments

MPP+ and rotenone were obtained from Sigma-Aldrich (Madrid, Spain). Stock solutions of 50 mM MPP+ in milliQ H_2_O and 10 mM rotenone in DMSO were freshly prepared on the day of the treatment. Cells were treated with 10 or 25 µM MPP+ or 40 or 100 nM rotenone for 24 h. The concentration of DMSO in the cell cultures was always below 1/1,000.

Cells were treated with 50 ng/mL IL4 for 24 h in the absence or presence of MPP+ or rotenone. Recombinant mouse IL4 expressed in CHO cells was used (Creative BioMart, Shirley, NY, USA). A stock solution of 50 mg/mL IL4 in a mixture of milli-Q H_2_O:culture medium (1:1) was prepared and stored at – 20 °C. A new aliquot was used in each experiment.

The agents were added directly to the culture medium.

### Propidium iodide and Hoechst staining

Propidium iodide and Hoechst labeling were performed on glial cells cultured in 96-well plates to determine cell death. Briefly, cells were incubated with propidium iodide (7.5 μg/mL; Invitrogen, Thermo Fisher Scientific, Waltham, MA, USA) for 10 min and Hoechst 33,342 (3 μg/mL; Invitrogen) for 20 min. Images were obtained with an Olympus IX70 microscope (Olympus, Okoya, Japan) and a digital camera (CC-12, Olympus Soft Imaging Solutions GmbH, Hamburg, Germany). Images from two wells/experimental condition were obtained with a 10X objective (mixed glial cultures) or a 4X objective (microglial cultures). The labeled nuclei were counted using ImageJ software. Cell death was measured as the ratio of the nuclei positive for propidium iodide, corresponding to dead cells, to the Hoechst-positive nuclei.

### RNA extraction and quantitative real-time PCR

The mRNA expression of anti-inflammatory markers was determined by quantitative real-time PCR 24 h after treatment in glial cells cultured in 6-well plates as described previously^23^. The High Pure RNA Isolation Kit (Roche Diagnostics Schweiz AG, Rotkreuz, Switzerland) was used to isolate total RNA from the mixed glial cultures (1 well/experimental condition), while the PureLink RNA Micro Scale Kit (Invitrogen) was used to isolate total RNA from the primary microglial cultures (2 wells/experimental condition). RNA (0.5–1 μg) was reverse transcribed with random primers using Transcriptor Reverse Transcriptase (Roche Diagnostics) and 3 ng of cDNA were used to perform qRT-PCR with SYBR Green Master Mix (PCR Biosystems, London, United Kingdom) in an iCycler My-IQ apparatus (Bio-Rad Laboratories, Hercules, CA, USA) as previously described (Dentesano et al., 2014). The primers used (Integrated DNA Technology, IDT, Skokie, IL, USA) are shown in Table 1. Relative gene expression values were calculated with the comparative Ct or ΔΔCt method ^64^. The genes encoding β-actin and Rn18s were used as the reference genes.

**Table 1:** Primers used for qRT-PCR.

Target mRNA	Accession number	Forward primer (5′ → 3′)	Reverse primer (5′ → 3′)
Arg1	NM_007482.3	TTGCGAGACGTAGACCCTGG	CAAAGCTCAGGTGAATCGGC
Carkl	NM_029031.3	CAGGCCAAGGCTGTGAAT	GCCAGCTGCATCATAGGACT
Cd200full	NM_010818.3	GGGCATGGCAGCAGTAGCG	TGTGCAGCGCCTTTCTTTC
Cd200tr	NM_001358443.1	GATGGGCAGTCTGTGGAAGTG	GAGAACATCGTAAGGATGCAGTTG
Cd200r1	NM_021325.3	AGGAGGATGAAATGCAGCCTTA	TGCCTCCACCTTAGTCACAGTATC
Fizz1	NM_020509.3	TCCCAGTGAATACTGATGAGA	CCACTCTGGATCTCCCAAGA
Glut1	NM_011400.3	CATCCTTATTGCCCAGGTGTTT	GAAGATGACACTGAGCAGCAGA
Hifα	NM_010431.2	ACAAGTCACCACAGGACAG	AGGGAGAAAATCAAGTCG
IL10	NM_010548.2	TGAATTCCCTGGGTGAGAAG	ACACCTTGGTCTTGGAGCTT
IL1ra	NM_031167.5	AGGCCCCACCACCAGCTTTGAGTC	TCACCCAGATGGCAGAGGCAACAA
MR	NM_008625.2	TCTTTTACGAGAAGTTGGGGTCAG	ATCATTCCGTTCACCAGAGGG
Pfkp	NM_019703.4	AAGCTATCGGTGTCCTGACC	TCCCACCCACTTGCAGAAT
Pgc1β	NM_133249.3	TCCAGAAGTCAGCGGCCT	CTGAGCCCGCAGTGTGG
TGFβ	NM_011577.2	TGCGCTTGCAGAGATTAAAA	AGCCCTGTATTCCGTCTCCT
Ym1	NM_009892.3	GGGCATACCTTTATCCTGAG	CCACTGAAGTCATCCATGTC
**Reference genes**			
β-Actin	NM_007393.5	CAACGAGCGGTTCCGATG	GCCACAGGATTCCATACCCA
Rn18s	NR_003278.3_	GTAACCCGTTGAACCCCATT	CCATCCAATCGGTAGTAGCG

*β-Actin* actin, beta, *Arg1* arginase1, *Carkl*, sedoheptulokinase; *Cd200full* CD200 antigen full length; *Cd200tr* CD200 antigen truncated; *Cd200r1* CD200 antigen receptor; *Fizz1* found in inflammatory zone 1; *Glut1* glucose transporter 1; *Hifα* hypoxia-inducible factor 1, alpha subunit; *IL10* interleukin 10; *IL1ra* interleukin 1 receptor antagonist; *MR* mannose receptor; *Pfkp* phosphofructokinase, platelet; *Pgc1β* peroxisome proliferative activated receptor gamma, coactivator 1 beta; *TGFβ* transforming growth factor, beta; *Rn18s* 18S ribosomal RNA.

### Total protein extraction and Western blot

Protein expression of the anti-inflammatory markers was determined by western blot 24 h after treatment, using glial cells cultured in 6-well plates. To isolate total protein, one well (mixed glial cultures) or a pool of two wells (microglial cultures) from a six-well plate was used for each experimental condition. After a cold wash with phosphate-buffered saline (PBS), cells were scraped and collected in 100 µL (mixed glia) or 25 μL (microglia) of RIPA buffer per well (1% Igepal CA-630, 5 mg/mL sodium deoxycholate, 1 mg/mL sodium dodecyl phosphate and the protease inhibitor cocktail Complete, Roche Diagnostics, in PBS). Samples were sonicated and centrifuged for 5 min at 10,400 g at 4 °C. The supernatant was collected and stored at − 20 °C. Protein concentration was determined using the Bio-Rad Protein Assay Kit (Bio-Rad Laboratories), based on the Bradford assay^65^.

Western blot analyses of total protein extracts were performed as described previously^43^. Briefly, 30 µg of protein were subjected to SDS-PAGE on a 10% polyacrylamide gel and then transferred to a polyvinylidene difluoride membrane (Millipore, Bedford, MA, USA). Membranes were incubated with goat polyclonal anti-arginase 1 (1:250; Santa Cruz Biotechnology, Inc., Heidelberg, Germany), rabbit polyclonal anti-mannose receptor (1:1,000; Abcam, Cambridge, UK) and mouse monoclonal anti-β-actin (1:40,000; Sigma-Aldrich) primary antibodies overnight at 4 °C. After repeated washing, the membranes were incubated with the corresponding horseradish peroxidase-conjugated rabbit anti-goat (1:2000; DAKO, Agilent, Santa Clara, CA, USA), donkey anti-rabbit (1:5,000; GE Healthcare Lifescience, Fisher Scientific, Madrid, Spain) and goat anti-mouse (1:5,000; Bio-Rad Laboratories) secondary antibodies for 1 h at room temperature. The signal was developed with the WesternBrith Sirius HRP substrate (Advansta, VWR, Barcelona, Spain) and images were obtained using a VersaDoc System (Bio-Rad Laboratories). Data are expressed as the ratio of the intensity of the band corresponding to the protein of interest to that corresponding to the loading control protein (β-actin).

### Phagocytosis

The phagocytic activity of microglia was assessed using microglial cultures grown in 48-well plates 24 h after treatment as described previously^11^. Briefly, cells were incubated for 1 h at 37 °C with fluorescent latex microspheres (FluoSpheres, carboxylate-modified microspheres, 2.0 μm, red fluorescence (580/605), 2% solids; Thermo Fisher Scientific) (1:1,000) 23 h after treatment. They were then washed three times with PBS and fixed with 4% paraformaldehyde for 15 min.

Immunocytochemistry was performed using a rabbit polyclonal anti-Iba1 primary antibody (1:500; Wako, Fujifilm Wako Chemicals, Richmond, USA), which is a specific marker for microglial cells. Cells were first incubated in 0.3% Triton-X-100 in PBS containing 1% bovine serum albumin (BSA) and 10% normal donkey serum for 20 min at room temperature, and then overnight at 4 °C with the primary antibody. Once rinsed in PBS, the cells were then incubated for 1 h at room temperature with the Alexa Fluor 488 donkey anti-rabbit secondary antibody (1:1,000; Invitrogen). Antibodies were diluted in 0.3% Triton X-100 in PBS containing 1% BSA and 10% normal donkey serum.

Images of three microscopic fields *using* a 20X objective were obtained with an Olympus IX70 fluorescence microscope (Olympus, Okoya, Japan) and a digital camera (CC-12). Two–three wells per experimental condition were processed and each experimental condition was repeated at least five times. FluoSpheres were visually counted and the number of phagocytic cells (cells with microspheres) and non-phagocytic cells (cells without microspheres), as well as the number of fluorescent microspheres per microglial cell were quantified. To further analyze the phagocytic activity of microglial cells under the different experimental conditions, we also classified the phagocytic cells into three groups according to their degree of phagocytic activity: (1) cells with fewer microspheres/cell than the mean value minus the variance in control cells, (2) cells with microspheres/cell in the range of the mean value ± variance in control cells and (3) cells with microspheres/cell higher than the mean value plus the variance in control cells.

### MTT assay

Metabolic activity was assessed with 3-(4,5-dimethylthiazol-2-yl)-2,5-diphenyl tetrazolium bromide (MTT) colorimetric assay, which was performed 24 h after treatment as described previously^11^. Briefly, MTT (Sigma-Aldrich) was added at a final concentration of 1 mg/mL to the glial cells seeded in 96-well plates. After incubation for 30 min (mixed glial cultures) or 90 min (microglial cultures) at 37 °C, the medium was removed and 200 μL of DMSO (Sigma-Aldrich) were added to each well. The optical density of the blue formazan formed was measured at 570 nm using a microplate reader (Multiskan Spectrum, Thermo Fisher Scientific, Vantaa, Finland). Readings at 650 nm were used to obtain the background levels. Results are expressed as percentages of the control.

### Intracellular ATP levels

Glial cells seeded in 96-well (mixed glia) or 6-well plates (microglia, two wells per experimental condition) were used to determine the intracellular levels of ATP using a luminescence assay kit (ATPlite Luminescence ATP Detection Assay System, PerkinElmer, Waltham, MA, USA) as described previously^11^. Briefly, cells were lysed 24 h after treatment and the ATP concentration was measured by quantifying the light produced from the reaction between the ATP and the added luciferase and D-luciferin. The emitted light was quantified using a luminometer (Orion Microplate Luminometer, Berthold Detection System, Germany). ATP concentrations in the samples were calculated from an ATP standard curve.

### Lactate release

Glial cells seeded in 24-well (mixed glia) or 6-well plates (microglia) were used to determine the extracellular levels of lactate by colorimetric detection using a Lactate Assay Kit (Sigma-Aldrich), following the manufacturer’s instructions. Briefly, culture media were collected 24 h after treatment and stored at − 80 °C. In addition, cells were lysed and the protein content was measured using the BCA Protein Assay Kit (Thermo Fisher Scientific). Lactate content was assayed in aliquots of culture media and the lactate concentrations were calculated from a lactate standard curve and expressed as nmol/µg protein.

### Data presentation and statistical analysis

The results are presented as mean and SEM. At least three independent experiments were performed for each analysis. Data were statistically analyzed with the GraphPad Prism software. The normality of the distribution of the data presented was confirmed using the D’Agostino and Pearson normality test. Statistical analyses were performed using the one-way analysis of variance (ANOVA) followed by the Newman-Keuls post-hoc test. For the statistical analysis of frequency distribution tables in the phagocytosis experiments Chi-square tests were performed. Values of p < 0.05 were considered statistically significant.

### Ethical statement

C57BL/6 mice were used in the present study. The experimental protocols used were approved by the Ethics and Scientific Committee of the University of Barcelona and CSIC (reference number OB-404/17). All the experiments were carried out in accordance with European Union directives (2010/63/EU) and Spanish regulations (Real Decreto 1386/2018) on the use of laboratory animals.

## Supplementary information

Supplementary information
