# Supplementary material for: Parkinsonian neurotoxicants impair the anti-inflammatory response induced by IL4 in glial cells: involvement of the CD200-CD200R1 ligand-receptor pair
Source: Sci Rep. 2020 Jun 30;10:10650. doi: 10.1038/s41598-020-67649-4 (PMC7326927; doi:10.1038/s41598-020-67649-4)
Supplement: Supplementary file 1 — Supplementary information [file 41598_2020_67649_MOESM1_ESM.docx]

PARKINSONIAN NEUROTOXICANTS IMPAIR THE ANTI-INFLAMMATORY RESPONSE INDUCED BY IL4 IN GLIAL CELLS: INVOLVEMENT OF THE CD200-CD200R1 LIGAND-RECEPTOR PAIR

Neus Rabaneda-Lombarte, Lucas Blasco-Agell, Joan Serratosa, Laura Ferigle, Josep Saura and Carme Solà

SUPPLEMENTARY INFORMATION

**
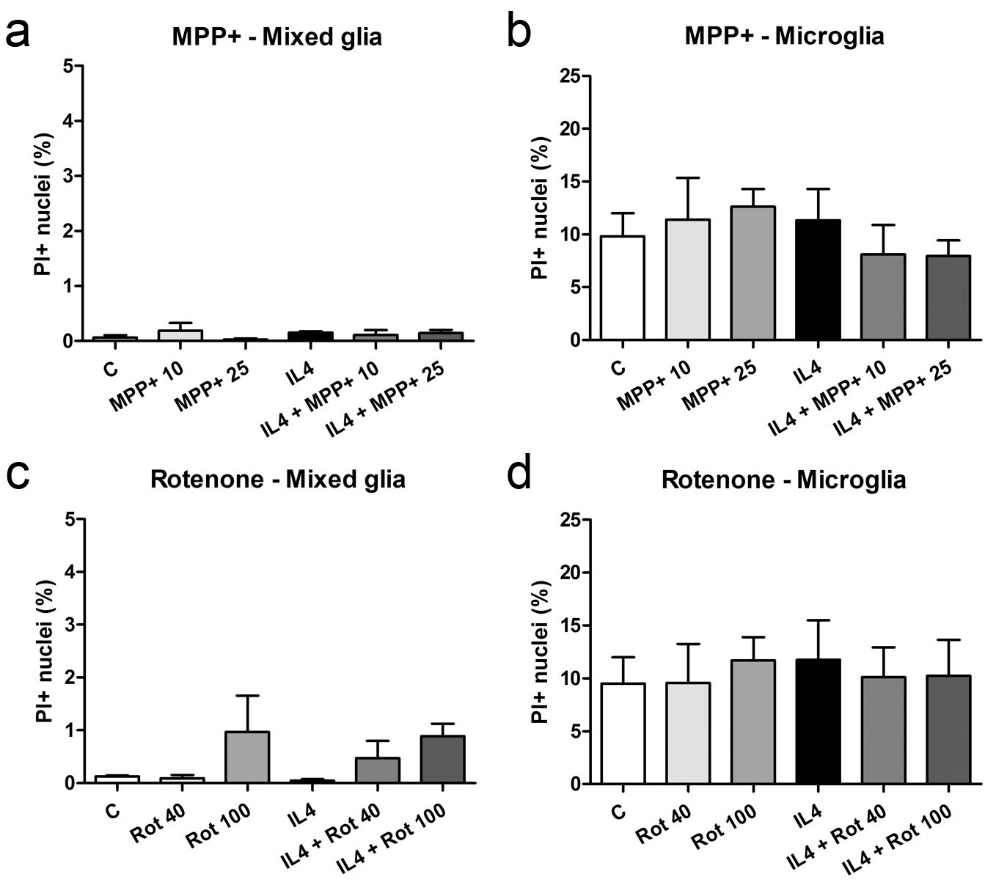
**

Supplementary Figure 1. Glial cell viability after MPP+ or rotenone treatment. (a-d) Percentage of propidium iodide (PI)-positive nuclei in mixed glial cultures (left column) and microglial cultures (right column) treated for 24 h with 10 or 25 μM MPP+ (a,b) or 40 or 100 nM rotenone (Rot) (c, d), both in the absence and presence of IL4 (50 ng/mL). Bars correspond to the means + SEM of five independent experiments. One-way ANOVA (repeated measures) p>0.05.
